# A cohort study examining emergency department visits and hospital admissions among people who use drugs in Ottawa, Canada

**DOI:** 10.1186/s12954-017-0143-4

**Published:** 2017-05-12

**Authors:** Claire E. Kendall, Lisa M. Boucher, Amy E. Mark, Alana Martin, Zack Marshall, Rob Boyd, Pam Oickle, Nicola Diliso, Dave Pineau, Brad Renaud, Tiffany Rose, Sean LeBlanc, Mark Tyndall, Olivia M. Lee, Ahmed M. Bayoumi

**Affiliations:** 1 Bruyère Research Institute, 43 Bruyère Street (Annex E), Ottawa, ON K1N 5C8 Canada; 2 Institute for Clinical Evaluative Sciences, Ottawa Hospital, Civic Campus, 1053 Carling Avenue, Box 684, Administrative Services Building, 1st Floor, Ottawa, ON K1Y 4E9 Canada; 3 Ottawa Hospital Research Institute, 1053 Carling Avenue, Ottawa, Ontario K1Y 4E9 Canada; 4 Social Development Studies and School of Social Work, Renison University College-University of Waterloo, 240 Westmount Road North, Waterloo, ON N2L 3G4 Canada; 5 Sandy Hill Community Health Centre, 221 Nelson Street, Ottawa, ON K1N 1C7 Canada; 6 Ottawa Public Health, 179 Clarence St, Ottawa, ON K1N 5P7 Canada; 7 PROUD Community Advisory Committee, Ottawa, ON Canada; 8 Drug Users Advocacy League, Ottawa, ON Canada; 9 BC Centre for Disease Control, 655W 12th Avenue, Vancouver, BC V5Z 4R4 Canada; 10 Faculty of Medicine, University of Ottawa, 451 Smyth Rd, Ottawa, ON K1H 8 L1 Canada; 11 Centre for Urban Health Solutions, Li Ka Shing Knowledge Institute and Division of General Internal Medicine, St. Michael’s Hospital, Toronto, Canada; 12 Department of Medicine and Institute of Health Policy, Management, and Evaluation, University of Toronto, Toronto ON 30 Bond St, Toronto, ON M5B 1 W8 Canada

**Keywords:** People who use drugs, Emergency department visits, Hospital admissions, Health administrative data, Self-reported data, Matched control group, Prescription drug benefits, Multimorbidity, Primary care, Opioid replacement therapy

## Abstract

**Background:**

The health of people who use drugs (PWUD) is characterized by multimorbidity and chronicity of health conditions, necessitating an understanding of their health care utilization. The objective of this study was to evaluate emergency department (ED) visits and hospital admissions among a cohort of PWUD.

**Methods:**

We used a retrospective observational design between 2012 and 2013. The population was a marginalized cohort of PWUD (the PROUD study) for whom survey data was linked (*n* = 663) to provincial health administrative data housed at the Institute for Clinical Evaluative Sciences. We constructed a 5:1 comparison group matched by age, sex, income quintile, and region. The main outcomes were defined as having two or more ED visits, or one or more hospital admissions, in the year prior to survey completion. We used multivariable logistic regression analyses to identify factors associated with these outcomes.

**Results:**

Compared to the matched cohort, PWUD had higher rates of ED visits (rate ratio [RR] 7.0; 95% confidence interval [95% CI] 6.5–7.6) and hospitalization (RR 7.7; 95% CI 5.9–10.0). After adjustment, factors predicting more ED visits were receiving disability (adjusted odds ratio [AOR] 3.0; 95% CI 1.7–5.5) or income assistance (AOR 2.7; 95% CI 1.5–5.0), injection drug use (AOR 2.1; 95% CI 1.3–3.4), incarceration within 12 months (AOR 1.6; 95% CI 1.1–2.4), mental health comorbidity (AOR 2.1; 95% CI 1.4–3.1), and a suicide attempt within 12 months (AOR 2.1; 95% CI 1.1–3.4). Receiving methadone (AOR 0.5; 95% CI 0.3–0.9) and having a regular family physician (AOR 0.5; 95% CI 0.2–0.9) were associated with lower odds of having more ED visits. Factors associated with more hospital admissions included Aboriginal identity (AOR 2.4; 95% CI 1.4–4.1), receiving disability (AOR 2.4; 95% CI 1.1–5.4), non-injection drug use (opioids and non-opioids) (AOR 2.2; 95% CI 1.1–4.4), comorbid HIV (AOR 2.4; 95% CI 1.2–5.6), mental health comorbidity (AOR 2.4; 95% CI 1.3–4.2), and unstable housing (AOR 1.9; 95% CI 1.0–3.4); there were no protective factors for hospitalization.

**Conclusions:**

Improved post-incarceration support, housing services, and access to integrated primary care services including opioid replacement therapy may be effective interventions to decrease acute care use among PWUD, including targeted approaches for people receiving social assistance or with mental health concerns.

## Background

People who use drugs habitually (PWUD) have multiple mental and physical health needs and a life expectancy 15 to 20 years shorter than that of the general population [1, 2]. Many PWUD do not receive longitudinal care from a primary care physician who could support the management of their addiction and comorbid conditions [3–5]. Instead, care for PWUD is often addiction-focused and episodic, including visits to emergency departments [6–9]. In a 2001 Vancouver, British Columbia study, 74% of people who inject drugs visited an emergency room over 39 months, and 60% of these individuals had three or more visits [6]. These visits often resulted in hospital admission [6]. The design and delivery of the health system has a profound impact on these health inequities [10]. Following Rhode’s concept of the “risk environment,” in which the interaction of the physical, structural, and social spaces contributes to harm among people who use drugs [11], people who are already socially marginalized, such as those living in poverty and Indigenous peoples, may be at highest risk for receiving poor health care.

A small number of studies using survey data, health records data, or both have identified the following predictors of greater emergency department use or hospital admissions: injecting crystal methamphetamine or cocaine, greater frequency of injection drug use, HIV-positive status, unstable housing, greater use of primary care, having an overdose, experiencing an assault, recent incarceration, receiving methadone, mental health issues, female sex, reporting being unable to obtain needed health care services, and having private health insurance [6, 12–15]. However, the majority of studies to date have been limited in their focus on subpopulations of PWUD, such as those who inject drugs, are on opioid replacement therapy or in other treatment [16–18], or are HIV positive [19–23], or on certain outcomes, such as infectious causes for acute care use [14, 24–26]. To the best of our knowledge, no studies have compared emergency department visits or hospital admissions among PWUD to those in the general community, nor made use of population-level administrative datasets to evaluate care for this population. An improved understanding of the rates of acute care use is critical for centres to anticipate care needs arising for PWUD, and given a large proportion of these visits may be preventable, an improved understanding of the determinants of these visits can identify potential areas for intervention [27]. In addition, given that drug use is an epidemic in evolution, in place, and in time, a contextual understanding of the characteristics of PWUD, and their implications for health services use is required to respond and adapt to drug policy environments.

The objectives of our study were to describe the rates of emergency department visits and hospital admissions by PWUD, compared to a matched population-based cohort and to determine the correlates of these encounters for this population. We used data from the Participatory Research in Ottawa: Understanding Drugs (PROUD) study [28], a community-based cohort study of PWUD in Ottawa, Canada, where rates of hepatitis C and HIV are among the highest of any major Canadian city [3]. We linked PROUD data to administrative databases, yielding a dataset with rich information about individual characteristics and health services use.

## Methods

### Setting and context

Among the 3500 to 600 people who use drugs in Ottawa, rates of Hepatitis C and HIV are among the highest of any major Canadian city Among Ottawa’s the 3500 to 6000 people who use drugs in Ottawa, rates of Hepatitis C and HIV are among the highest of any major Canadian city [3, 29]. An estimated 2263 area residents take opioid agonist therapy [30], and among those on opioid agonist treatment in the province of Ontario in 2014/15, about 80% took methadone [31]. Wait times for substance use treatment services is variable, with an average wait time from assessment to starting treatment of 29 days (source Ottawa Addictions Assessment and Referral Service, unpublished). Our region does not currently have supervised injection services.

### Participants

The PROUD study has been described previously [28]. Briefly, we used peer-guided street-based recruitment using snowball sampling to enrol participants in a cross-sectional survey study, focusing on socially and economically marginalized PWUD. Eligibility criteria included an age of at least 16 years and self-reported use of an illicit drug use other than marijuana by any route in the 12 months prior to the enrolment (March to December 2013). The survey was interviewer-administered and included questions about socio-demographic information, drug use, interpersonal variables (e.g., sexual history, community integration), environmental-structural variables (e.g., harm reduction, housing, legal matters), and health and health services use. Participants received a cash honoraria of $20 Canadian for participation in the study. All PROUD activities were governed by a Community Advisory Committee of PWUD and allies.

Participants were also asked to consent to link their survey data to the administrative databases held at the Institute for Clinical Evaluative Sciences (ICES). ICES databases are made available to accredited researchers through a data sharing agreement with the Ontario Ministry of Health and Long-Term Care. The PROUD and ICES datasets were linked either deterministically, using unique, encoded identifiers derived from participants’ reported Ontario Health Insurance Plan numbers, or probabilistically (when insurance numbers were unavailable) based on participants’ names, dates of birth, and postal codes. Participants with duplicate enrolment were identified following linkage; the record with the most complete data was retained.

We used the following ICES databases: Registered Persons Database (demographic and mortality data for all residents eligible for provincial health care), Ontario Health Insurance Program (OHIP) billing claims system (about 95% of physician services in Ontario), Community Health Centre database (encounter information for visits to Community Health Centres), Discharge Abstract Database (all hospital admission and discharge data), National Ambulatory Care Reporting System (emergency department visits), Client Agency Program Enrolment Registry (patient enrolment with individual primary care physicians), ICES Physician Database (physician demographic information, training, and practice setting), Corporate Provider Database (physician and group level data), Ontario Mental Health Reporting System (all admissions to designated mental health beds), Same Day Surgery database (all same day surgeries), CONTACT (eligibility summaries and yearly health services contact), Ontario Drug Benefits (prescription claims for individuals covered by the public system including those aged 65 and older and those receiving support from the Ontario Disability Support Program, Ontario Works (income assistance) and Trillium (a provincial catastrophic drug insurance program), Drug Identification Number database (drug list from Ontario Drug Benefits formularies, including generic names, trade names, and strengths), and Ontario HIV database (an ICES-derived cohort). We also used 2006 Statistics Canada Census data to infer income quintile by linking postal code of residence to the mean household income by dissemination area, which represents a standard geographic area typically consisting of 400 to 700 individuals.

To compare PROUD participants to the general population, we randomly selected control individuals, matched on age, sex, public health unit, and income quintile (using postal code) in a 5:1 ratio [32].

### Variable definitions

We categorized gender using self-reported data in the PROUD survey except when gender was missing or when participants reported gender as “two-spirited” or “other”, in which case we used ICES data (sex at birth). We excluded transgender individuals (<6). We used postal code to assign neighborhood income into quintiles. We classified comorbidity using the Johns Hopkins Adjusted Clinical Groups Case-Mix Assignment software (Sun Microsystems Inc., Santa Clara, CA) by assigning up to 32 distinct Aggregated Diagnosis Groups (ADGs) [33]. We categorized comorbidity as low (≤5 ADGs), medium (6–9 ADGs), or high (≥10 ADGs), and we used validated ICES algorithms to classify the presence of mental health diagnoses and HIV [34, 35].

Ontario has several mechanisms of prescription drug benefits (Ontario Drug Benefits), including coverage for those aged 65 and older and those receiving support from the Ontario Disability Support Program, Ontario Works (income assistance), and Trillium (a provincial catastrophic drug insurance program). Ontario has distinct models of primary care with different reimbursement mechanisms, such as capitation with rostering of patients to physicians and organizational structures, such as the presence of interprofessional teams. We categorized primary care models according to whether they were community health centres or conventional practices, team-based or not, and whether reimbursement was based on capitation, fee for service payments, or enhanced fee for service [36]. Rostered participants were assigned to their primary care physician; non-rostered participants were assigned to the family physician who provided the majority of the costs of their primary care in the year prior to enrolment. We counted the number of primary care visits excluding visits that were exclusively for methadone therapy.

The majority of variables arising from PROUD survey data were dichotomized to yes versus no, with the no category including any non-yes response (including don’t know/unsure, no answer, and missing responses, with up to 25 participants providing don’t know/unsure responses, and up to 30 participants providing no answer responses). Missing responses usually occurred on sub-questions due to skip patterns dictated by the responses to parent questions (and when the missing responses were not due to skipped sub-questions, they occurred for up to 20 participants across questions).

### Outcomes

Our co-primary outcome measures were emergency department visits and hospital admissions in the year prior to enrolment after excluding maternity-related admissions and same-day surgeries. We categorized emergency department visits by acuity level using the Canadian Triage and Acuity Score (CTAS) from 1 (highest) to 5 (lowest). We ascertained diagnoses using the most responsible diagnosis for emergency department visits, and any diagnosis for hospital admission diagnoses.

### Analyses

Comparative rates of emergency department visits and hospital admissions (number of events per year) between the PROUD participants and the matched cohort were stratified by gender and analyzed using chi-squared or Fisher’s exact test for categorical variables and Wilcoxon rank sum tests for continuous variables. We used logistic regression to analyze variables associated with having two or more emergency department visits and to analyze variables associated with one or more hospital admission; we conducted these analyses both comparing PROUD participants to the matched cohort and within the PROUD cohort alone. We used a non-parsimonious approach to selecting covariates but excluded those that we judged likely to be collinear. Cell sizes of 6 or less are reported in aggregate only to preserve privacy. All statistical analyses were conducted using SAS statistical software, version 9.3 (SAS Institute Inc., Cary, NC). This study was approved by the institutional review board at Sunnybrook Health Sciences Centre, Toronto, Canada, and the Ottawa Health Sciences Network Research Ethics Board (OHSN-REB #20120566-01H).

## Results

Of 858 PROUD participants, 798 agreed to data linkage. We excluded participants without Ontario health insurance and those who were likely duplicate enrolments. Of the remaining 782 participants, 663 (85%) were successfully linked. Among the analysis cohort, the median age was 41.4 years, 75.6% were male, 66.7% were in the lowest two income quintiles, and 78.3% received disability payments or income assistance (Table 1). Over half of PROUD participants had a mental health-related diagnosis other than substance use-related care. About equal proportions of PROUD participants and matched cohort individuals had a primary care physician but PROUD participants were more likely to receive care in a community health centre and had about three times as many primary care visits.

**Table 1:** Characteristics of PROUD participants (*n* = 663), and an Ontario population (*n* = 3,315) matched by age, sex, public health unit, and neighborhood income quintile

Characteristic		PROUD (*N* = 663) (*n*/*N*, (%))	Matched cohort (*N* = 3315) (*n*/*N*, (%))
Age		41.4 (10.8)	41.0 (10.8)
Age category	<=24	54 (8.1)	291 (8.8)
	25 to 34	134 (20.2)	676 (20.4)
	35 to 44	182 (27.5)	937 (28.3)
	45 to 54	229 (34.5)	1126 (34)
	> = 55	64 (9.7)	285 (8.6)
Sex^a^	Male	501 (75.6)	2505 (75.6)
	Female	162 (24.4)	810 (24.4)
Local Health Integration Network	Champlain	610 (92)	3315 (100)
	Other	35 (5.3)	
	Out of province/missing	18 (2.7)	
Income quintile	1 (lowest)	246 (37.1)	1230 (37.1)
	2	196 (29.6)	980 (29.6)
	3	144 (21.7)	720 (21.7)
	4	36 (5.4)	180 (5.4)
	5 (highest)	28 (4.2)	169 (5.1)
	Missing	13 (2)	36 (1.1)
Prescription drug benefits	Ontario Works	164 (24.7)	111 (3.4)
	Ontario Disability Support Program	342 (51.6)	213 (6.4)
	Other, including no coverage	157 (23.7)	2991 (90.2)
Comorbidity (# of aggregated diagnosis groups (ADGs) in 2 year prior to cohort entry)	Low comorbidity (0–5 ADGs)	273 (41.2)	2048 (61.8)
	Medium comorbidity (6–9 ADGs)	220 (33.2)	1072 (32.3)
	High comorbidity (> = 10 ADGs)	170 (25.6)	195 (5.9)
Comorbid mental health conditions (excluding substance use diagnoses)		362 (54.6)	595 (18)
Comorbid HIV		50 (7.5)	16 (0.5)
Has a regular family physician		542 (81.8)	2915 (87.9)
Primary care model	Team-based capitation	103 (15.5)	522 (15.8)
	Non-team-based capitation	61 (9.2)	894 (27)
	Enhanced fee for service	223 (33.6)	1177 (35.5)
	Traditional fee for service	68 (10.3)	260 (7.8)
	Community health centre	87 (13.1)	62 (1.9)
	Orphan patients (unrostered)	121 (18.3)	400 (12.1)
# of outpatient primary care visits in 1 year prior to survey completion (non-methadone)		10.2 (16.9)	3.2 (5.1)

^a^We categorized gender using self-reported data in the PROUD survey except when gender was missing or when participants reported gender as “two-spirited” or “other”, in which case we used ICES data (sex at birth). We excluded transgender individuals (<6)

Compared to the matched cohort, PROUD participants had a significantly higher rate of emergency department visits (2.1 vs. 0.3 visits per year; rate ratio [RR] 7.0; 95% confidence interval [95% CI] 6.5 to 7.6) (Table 2). The rate ratio was similar among PROUD participants when we restricted analyses to men, women, and people with higher acuity visits (CTAS 1, 2, or 3). PROUD participants were much more likely than controls to visit an emergency room for a mental health-related diagnosis, including both substance use-related visits (RR 150.0; 95% CI 86.3 to 260.7) and other mental health visits (RR 16.0; 95% CI 10.3 to 24.8). Rates among PROUD participants were also significantly higher than controls for infectious diseases (RR 12.0; 95% CI 8.1 to 17.8), including soft tissue infection and pneumonia. In contrast to the matched cohort (from whom the number of visits were too few to report), PROUD participants also had measurable rates of cocaine-related visits (9.2 visits per 100 person-years), visits for overdose (4.7 visits per 100 person-years), self-harm (4.5 visits per 100 person-years), and opioid use (2.4 visits per 100 person-years).

**Table 2:**
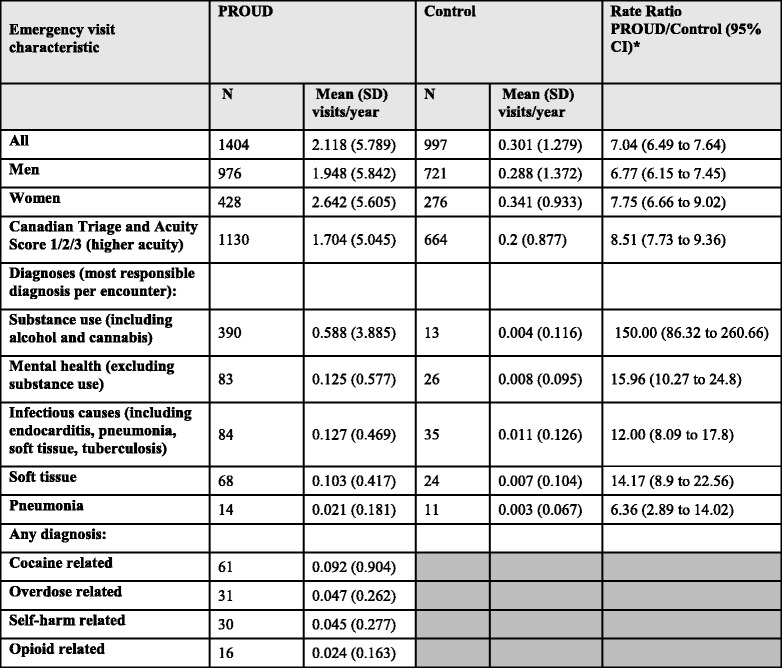
Rates of emergency department visits among PROUD participants (*n* = 663) compared to an Ontario population (*n* = 3315) matched by age, sex, public health unit, and neighborhood income quintile

*Shaded cells reflect cases < = 6 participants

Compared to the matched cohort, PROUD participants had a significantly higher rate of hospital admissions (RR 7.7; 95% CI 5.9 to 10.0) (Table 3). Rates were higher for women than for men (30.9 visits per 100 person-years vs. 18.2, respectively). However, the rate ratios comparing PROUD participants to individuals in the matched cohort were similar for men (7.2) and women (8.6). Among PROUD participants, the most common reasons for hospital admission were substance use (7.7 admissions per 100 person-years), mental health excluding substance use (4.4 admissions per 100 person-years), and infectious causes (4.4 admissions per 100 person-years); pneumonia and soft tissue infections accounted for most of the infection-related admissions.

**Table 3:** Rates of hospital admissions among PROUD participants (*n* = 663) compared to an Ontario population (*n* = 3315) matched by age, sex, public health unit, and neighborhood income quintile

Hospital admission characteristic	PROUD		Control		Rate ratio PROUD/control (95% CI)
	*N*	Mean (SD) visits/year	*N*	Mean (SD) visits/year	
All	141	0.213 (0.608)	92	0.028 (0.249)	7.66 (5.89 to 9.97)
Males	91	0.182 (0.549)	63	0.025 (0.187)	7.22 (5.24 to 9.96)
Females	50	0.309 (0.758)	29	0.036 (0.382)	8.62 (5.46 to 13.62)
Diagnoses (any diagnosis per encounter)					
Substance use (including alcohol and cannabis)	51	0.077 (0.304)	9	0.003 (0.052)	28.33 (13.95 to 57.55)
Mental health (excluding substance use)	29	0.044 (0.274)	7	0.002 (0.052)	20.71 (9.07 to 47.29)

After adjusting for HIV status, mental health diagnosis, receipt of disability or income assistance, and linkage with primary care, PROUD participants were still much more likely than individuals from the matched cohort to have two or more emergency department visits (adjusted odds ratio [AOR] 3.3; 95% CI 2.4 to 4.7), or to have one or more hospital admissions (AOR 2.2; 95% CI 1.4 to 3.6) (Table 4).

**Table 4:** Multivariable logistic regression of PROUD participation on 2+ emergency department visits and 1+ hospital admission, adjusted for listed covariates

Covariate		2+ ED visits AOR (95% CI)	2+ low acuity ED visits AOR (95% CI)	2+ high acuity ED visits AOR (95% CI)	1+ hospital admission AOR (95% CI)
PROUD participant	Yes	3.33 (2.38 to 4.66)	1.42 (0.79 to 2.57)	4.53 (3.04 to 6.76)	2.24 (1.41 to 3.55)
	No	Ref	Ref	Ref	Ref
Comorbid HIV	Yes	2.67 (1.06 to 6.76)	0.40 (0.07 to 2.14)	5.44 (1.52 to 19.49)	2.58 (0.82 to 8.09)
	No	Ref	Ref	Ref	Ref
Comorbid mental health diagnosis (excluding substance use-related diagnoses)	Yes	2.67 (1.96 to 3.64)	2.77 (1.55 to 4.96)	3.56 (2.4 to 5.26)	2.37 (1.46 to 3.86)
	No	Ref	Ref	Ref	Ref
Prescription drug benefits (any)	Yes	2.75 (1.90 to 3.98)	7.34 (3.51 to 15.31)	2.56 (1.61 to 4.07)	4.72 (2.70 to 8.23)
	No	Ref	Ref	Ref	Ref
Has a regular family physician	Yes	0.26 (0.15 to 0.47)	0.66 (0.24 to 1.84)	0.22 (0.11 to 0.44)	0.64 (0.32 to 1.3)
	No	Ref	Ref	Ref	Ref

*AOR* adjusted odds ratio

When we analyzed only PROUD participants, after adjustment, the strongest independent associations with emergency department use, classified as two or more visits, were receiving disability payments (AOR 3.0; 95% CI 1.7 to 5.5) or income assistance (AOR 2.7; 95% CI 1.5 to 5.0), any injection drug use in the previous 12 months (AOR 2.1; 95% CI 1.3 to 3.4), incarceration in the previous 12 months (AOR 1.6; 95% CI 1.1 to 2.4), mental health comorbidity (AOR 2.1; 95% CI 1.4 to 3.1), and having a suicide attempt in the previous 12 months (AOR 2.1; 95% CI 1.1 to 3.4) (Table 5). Receiving methadone (AOR 0.5; 95% CI 0.3 to 0.9) and having a regular family physician (AOR 0.5; 95% CI 0.2 to 0.9) were associated with lower odds of having two or more emergency department visits. In a similar adjusted analysis of one or more hospital admissions, the strongest associations were with self-identified Aboriginal identity (AOR 2.4; 95% CI 1.4 to 4.1), receiving disability payments (AOR 2.4; 95% CI 1.1 to 5.4), non-injection drug use (both opioids and non-opioids) (AOR 2.2; 95% CI 1.1 to 4.4), comorbid HIV (AOR 2.4; 95% CI 1.2 to 5.6), mental health comorbidity (AOR 2.4; 95% CI 1.3 to 4.2), and unstable housing (AOR 1.9; 95% CI 1.0 to 3.4). No factors were associated with lower odds of having one or more hospital admissions.

**Table 5:** Multivariable logistic regression of PROUD participant characteristics associated with 2+ emergency department visits or 1+ hospital admission, adjusted for listed covariates

Variable		2+ ED visits AOR (95% CI)	1+ hospital admission AOR (95% CI)
Age (per year)		0.98 (0.96 to 1.00)	0.99 (0.97 to 1.02)
Gender	Male	Ref	Ref
	Female	1.37 (0.86 to 2.20)	1.57 (0.88 to 2.79)
Ethnicity	Aboriginal	1.58 (0.98 to 2.55)	2.39 (1.38 to 4.13)
	Other	Ref	Ref
Sexual orientation	Straight	Ref	Ref
	Gay/lesbian/homosexual/other	1.39 (0.77 to 2.51)	0.54 (0.25 to 1.18)
Neighborhood of residence	Market/lowertown	1.05 (0.66 to 1.68)	1.12 (0.63 to 1.98)
	Centretown	0.36 (0.2 to 0.65)	0.45 (0.2 to 1.01)
	Other	Ref	Ref
Income quintile	1 and missing (lowest)	1.3 (0.66 to 2.53)	1.13 (0.48 to 2.67)
	2	1.06 (0.53 to 2.14)	0.91 (0.37 to 2.21)
	3	0.81 (0.39 to 1.68)	1.36 (0.55 to 3.35)
	4 and 5 (highest)	Ref	Ref
Highest level of education	Some HS or less	1.22 (0.61 to 2.44)	1.07 (0.44 to 2.6)
	High school graduate or equivalent	1.44 (0.7 to 2.93)	1.16 (0.46 to 2.92)
	Some college or university	2.14 (0.99 to 4.61)	1.72 (0.67 to 4.47)
	College or university completed	Ref	Ref
Prescription drug benefits	Ontario Disability Support Program	3.00 (1.65 to 5.45)	2.41 (1.08 to 5.39)
	Ontario Works	2.74 (1.5 to 5.02)	2.15 (0.95 to 4.89)
	Other, including no coverage	Ref	Ref
Sex work as primary source of income	Yes	0.52 (0.25 to 1.09)	1.03 (0.43 to 2.45)
	No	Ref	Ref
Housing	Unstable	1.52 (0.96 to 2.42)	1.85 (1.02 to 3.38)
	Stable	Ref	Ref
Detained in jail overnight or longer in the last 12 months	Yes	1.62 (1.08 to 2.43)	0.97 (0.58 to 1.6)
	No	Ref	Ref
Drug use in past 12 months	Any injection	2.08 (1.26 to 3.43)	1.51 (0.79 to 2.88)
	Non-injection drug use (both opioids and non-opioids)	1.51 (0.87 to 2.62)	2.21 (1.12 to 4.37)
	Non-injection use of only non-opioids	Ref	Ref
Overdosed in past 12 months	Yes	1.3 (0.79 to 2.14)	1.37 (0.76 to 2.47)
	No	Ref	Ref
Comorbid HIV	Yes	1.57 (0.79 to 3.11)	2.54 (1.16 to 5.55)
	No	Ref	Ref
Last test for HCV result was positive	Yes	0.91 (0.58 to 1.43)	0.97 (0.55 to 1.71)
	No	Ref	Ref
Comorbid mental health diagnosis (excluding substance use-related diagnoses)	Yes	2.06 (1.35 to 3.14)	2.36 (1.31 to 4.24)
	No	Ref	Ref
Attempted suicide in last 12 months	Yes	2.08 (1.13 to 3.83)	1.91 (0.97 to 3.74)
	No	Ref	Ref
Currently on methadone	Yes	0.54 (0.34 to 0.88)	1.15 (0.64 to 2.08)
	No	Ref	Ref
Accessed addiction treatment in past 12 months	Yes	1 (0.67 to 1.5)	0.97 (0.58 to 1.6)
	No	Ref	Ref
Has a regular family physician	Yes	0.45 (0.23 to 0.86)	1.02 (0.45 to 2.33)
	No	Ref	Ref

*AOR* adjusted odds ratio

## Discussion

We used a combination of rich self-reported data and robust health administrative data to assess the use of acute care services among PWUD. Our main finding is that PWUD continue to use emergency and hospital services at disproportionately high rates compared to the general population (approximately seven to eight times more frequently) and that most of this use is related to drug use and other mental health-related problems. Our results underscore the significant burden of mental health illness experienced by PWUD, and the ongoing need for comprehensive and continuing mental health care. Whether such supports can avert use of acute care services is a topic for future research.

We found that receiving disability or income assistance were associated with increased use of emergency departments or hospital admissions. Participants who were HIV-positive were more likely to be hospitalized. Aboriginal ethnicity was also associated with increased hospitalization. These findings likely relate to the significant and synergistic effects of poverty, structural racism, and comorbidities on individuals’ health and the incomplete amelioration of these effects by current public assistance programs [37, 38].

We identified two factors that may be important in averting use of acute care services. Receipt of methadone was associated with an approximately 50% lowered risk of visiting an emergency department at least twice in a year. Having a regular family physician was associated with a similar reduction in emergency department visits. In contrast to some findings [12, 39], our study supports linkage to a regular source of primary care to optimize the health of PWUD [40]. We found that having unstable housing was associated with an almost twofold increased risk of hospitalization; programs that target people who are homeless have been shown to reduce the number of hospitalizations, the length of hospital stays, and the number of emergency department visits [41].

Being detained in jail overnight or longer in the last 12 months was associated with higher odds of having two or more emergency department visits. To date, Ottawa has had a prohibitionist drug policy environment: people in our cohort have experienced negative interactions with police arising from their drug use that has led to frequent incarcerations, with 77% of our cohort having had spent one or more nights in jail in the year prior to survey completion and 30% experiencing “red zoning”, the geographic restriction of access to certain areas of the city by police. Transitioning from incarceration is a highly destabilized period, contributing to greater emergency department use and poor linkages to primary care despite a high prevalence of chronic disease in this population [42, 43]. Furthermore, this association also speaks to the contribution of the criminal justice system in perpetuating harms among people who use drugs [11].

A strength of our study is the use of community-based participatory research methods to obtain survey data on a highly disadvantaged population, and the use of population-level data to characterize health care use in a setting with universal health insurance. However, our study has limitations. The PROUD survey relied on self-reported data which may be prone to social desirability or other reporting biases. Participants were asked about practices that are highly stigmatized or illegal, which may contribute to underreporting of high-risk practices. We used a street-based peer recruitment approach to reach “hidden populations” in order to improve representativeness over standard recruitment methods [28, 44], however, it is possible that our findings are not widely generalizable. PROUD was a cross-sectional study so we are unable to look at trends over time, including changes in drug use. Finally, ICES data are collected for administrative rather than research purposes. Social variables such as income are ascertained using neighborhood-level metrics (postal codes). However, linkage to PROUD survey data improved the detail of social level variables available for our population.

## Conclusions

In conclusion, our study quantified the substantial use of acute care services among PWUD, in particular for mental health and addiction-related issues. Our findings underscore the intersection of multi-level, social-structural factors [45] that influence health care use among PWUD, such as mental health and physical health comorbidity, poverty, social instability, structural racism, and the persistent criminalisation of drug use. Future research should highlight the potential role for integrated primary care and housing services and stability in mitigating this disparity in service use among PWUD. In addition, we recommend interventions to improve linkage to care post-incarceration among PWUD, and explicit evaluations of the impact of the criminal justice system on health care utilization.

## Abbreviations

ADG: Aggregated Diagnosis Groups
CTAS: Canadian Triage and Acuity Score
HIV: Human immunodeficiency virus
ICES: Institute for Clinical Evaluative Sciences
OHIP: Ontario Health Insurance Program
OR: Odds ratio
PROUD: Participatory Research in Ottawa: Understanding Drugs
PWUD: People who use drugs
RR: Rate ratio
