# Erratum to: A cohort study examining emergency department visits and hospital admissions among people who use drugs in Ottawa, Canada

**DOI:** 10.1186/s12954-017-0169-7

**Published:** 2017-07-03

**Authors:** Claire E. Kendall, Lisa M. Boucher, Amy E. Mark, Alana Martin, Zack Marshall, Rob Boyd, Pam Oickle, Nicola Diliso, Dave Pineau, Brad Renaud, Tiffany Rose, Sean LeBlanc, Mark Tyndall, Olivia M. Lee, Ahmed M. Bayoumi

**Affiliations:** 1 Bruyère Research Institute, 43 Bruyère Street (Annex E), Ottawa, ON K1N 5C8 Canada; 2 Institute for Clinical Evaluative Sciences, Ottawa Hospital, Civic Campus, 1053 Carling Avenue, Box 684, Administrative Services Building, 1st Floor, Ottawa, ON K1Y 4E9 Canada; 3 Ottawa Hospital Research Institute, 1053 Carling Avenue, Ottawa, Ontario K1Y 4E9 Canada; 4 Social Development Studies and School of Social Work, Renison University College-University of Waterloo, 240 Westmount Road North, Waterloo, ON N2L 3G4 Canada; 5 Sandy Hill Community Health Centre, 221 Nelson Street, Ottawa, ON K1N 1C7 Canada; 6 Ottawa Public Health, 179 Clarence St, Ottawa, ON K1N 5P7 Canada; 7 PROUD Community Advisory Committee, Ottawa, ON Canada; 8 Drug Users Advocacy League, Ottawa, ON Canada; 9 BC Centre for Disease Control, 655W 12th Avenue, Vancouver, BC V5Z 4R4 Canada; 10 Faculty of Medicine, University of Ottawa, 451 Smyth Rd, Ottawa, ON K1H 8 L1 Canada; 11 Centre for Urban Health Solutions, Li Ka Shing Knowledge Institute and Division of General Internal Medicine, St. Michael’s Hospital, Toronto, Canada; 12 Department of Medicine and Institute of Health Policy, Management, and Evaluation, University of Toronto, Toronto ON 30 Bond St, Toronto, ON M5B 1 W8 Canada

## Erratum

Upon publication of the original article [1], it was noticed that an error occurred at the beginning of the Methods section. This originally appeared as:

“Among the 3500 to 600 people who use drugs in Ottawa, rates of Hepatitis C and HIV are among the highest of any major Canadian city Among Ottawa’s the 3500 to 6000 people who use drugs in Ottawa, rates of Hepatitis C and HIV are among the highest of any major Canadian city [3]”

This has now been updated to:

“Among the 3500 to 6000 people who use drugs in Ottawa, rates of Hepatitis C and HIV are among the highest of any major Canadian city [3].”
